# Comparison of improved range of motion between cam-type femoroacetabular impingement and borderline developmental dysplasia of the hip -evaluation by virtual osteochondroplasty using computer simulation-

**DOI:** 10.1186/s12891-017-1778-8

**Published:** 2017-10-16

**Authors:** So Kubota, Yutaka Inaba, Naomi Kobayashi, Hyonmin Choe, Taro Tezuka, Tomoyuki Saito

**Affiliations:** Department of Orthopaedic Surgery, Yokohama City University, 3-9Fukuura, Kanazawa-ku, Yokohama, Kanagawa 236-0004 Japan

**Keywords:** Femoroacetabular impingement, Borderline developmental dysplasia of the hip, Impingement simulation, Range of motion

## Abstract

**Background:**

While cam resection is essential to achieve a good clinical result with respect to femoroacetabular impingement (FAI), it is unclear whether it should also be performed in cases of borderline developmental dysplasia of the hip (DDH) with a cam deformity. The aim of this study was to evaluate improvements in range of motion (ROM) in cases of cam-type FAI and borderline DDH after virtual osteochondroplasty using a computer impingement simulation.

**Methods:**

Thirty-eight symptomatic hips in 31 patients (11male and 20 female) diagnosed with cam-type FAI or borderline DDH were analyzed. There were divided into a cam-type FAI group (cam-FAI group: 15 hips), borderline DDH without cam group (DDH W/O cam group: 12 hips), and borderline DDH with cam group (DDH W/ cam group: 11 hips). The bony impingement point on the femoral head-neck junction at 90° flexion and maximum internal rotation of the hip joint was identified using ZedHip® software. Virtual osteochondroplasty of the impingement point was then performed in all cases. The maximum flexion angle and maximum internal rotation angle at 90° flexion were measured before and after virtual osteochondroplasty at two resection ranges (i.e., slight and sufficient).

**Results:**

The mean improvement in the internal rotation angle in the DDH W/ cam group after slight resection was significantly greater than that in the DDH W/O cam group (*P* = 0.046). Furthermore, the mean improvement in the internal rotation angle in the DDH W/ cam and cam-FAI groups after sufficient resection was significantly greater than that in the DDH W/O cam group (DDH W/ cam vs DDH W/O cam: *P* = 0.002, cam-FAI vs DDH W/O cam: *P* = 0.043).

**Conclusion:**

Virtual osteochondroplasty resulted in a significant improvement in internal rotation angle in DDH W/ cam group but not in DDH W/O cam group. Thus, borderline DDH cases with cam deformity may be better to consider performing osteochondroplasty.

## Background

Femoroacetabular impingement (FAI) is an important cause of hip pain and subsequent osteoarthritis [1]. It is caused by an anatomical abnormality that results in mechanical impingement between the acetabular rim and the femoral head-neck junction during flexion and internal rotation of the hip [2, 3]. A major factor is cam deformity of the femur, which is characterized by an aspherical femoral head and bony bump formation at the femoral head-neck junction, which reduces femoral head-neck offset [2, 3]. FAI causes hip pain during squatting or deep flexion and may therefore reduce range of motion (ROM) at that joint [4]. These morphological abnormalities can be corrected by osteochondroplasty, which releases the bony impingement and improves ROM.

Developmental dysplasia of the hip (DDH) also causes hip pain, and has an etiological factor in the development of hip osteoarthritis [5, 6]. Its essential concept is that a biomechanical abnormality, joint incongruity, or decreased joint contact area may increase mechanical stress at the acetabular rim [6]. Although the basic pathophysiology of DDH is different from that of FAI, there are cases in which borderline DDH co-exists with a cam deformity [7, 8]. While cam resection is essential to achieve a good clinical result with respect to FAI [9, 10], it is unclear whether it should also be performed in cases of borderline DDH. In this regard, computer simulated virtual osteochondroplasty may provide the answer from the point of view of improvement in ROM.

The aim of this study was to investigate improvement in ROM after virtual hip osteochondroplasty using computer impingement simulation models for cam type FAI, borderline DDH with cam deformity, and borderline DDH without cam deformity.

We hypothesized that borderline DDH with cam deformity and cam-type FAI cases would demonstrate similar and significant improvement in ROM after virtual osteochondroplasty.

## Methods

This study was approved by the institutional review board at Yokohama City University. In total, 38 symptomatic hips in 31 patients (11 male and 20 female) diagnosed with cam-type FAI or borderline DDH by plain radiography were enrolled. The mean age of the patients was 47.8 ± 13.0 years (range, 21–74 years). The anterior impingement test was positive in all 38 hips. Computed tomography (CT) and radiographic evaluation were performed during the same period (within 3 months) in all cases.

### Radiographic evaluation

The lateral center-edge (CE) angle, Tönnis grade and crossover sign were measured on an antero-posterior (A-P) view of the pelvis. The alpha angle was measured on a cross table lateral view of the hip joint. The measurement procedure was standardized in the picture archiving and communication system.

### Definition of cam-type FAI and borderline DDH

Cam-type FAI was defined as an alpha angle ≥ 55° [11, 12] and a CE angle ≥ 25° [13, 14]. Borderline DDH was defined as a CE angle between 20 and 24° [13, 15]. Borderline DDH with cam deformity was defined as a combination of borderline DDH (20 ≤ CE angle < 25°) and cam (alpha angle ≥ 55°). Thus, the 38 hips were assigned to three groups: a cam-type FAI group (cam-FAI group; 15 hips), a borderline DDH without cam deformity group (DDH W/O cam group; 12 hips), and a borderline DDH with cam deformity group (DDH W/ cam group; 11 hips). Pincer-type FAI cases (CE angle > 40°) [16], DDH with a CE angle of < 20°, or osteoarthritic changes with a Tönnis grade ≥ 2 were excluded.

### Computed tomography

CT scans (Sensation16; Siemens AG, Erlangen, Germany) of the pelvis and femurs of all patients were acquired using the following scanner settings: 140 kV and 300 mA; slice thickness, 1.5 mm; pixel resolution, 512 × 512; voxel size, 0.70 × 0.70 × 1.5 mm.

### Computer simulated impingement analysis

**Fig. 1:**
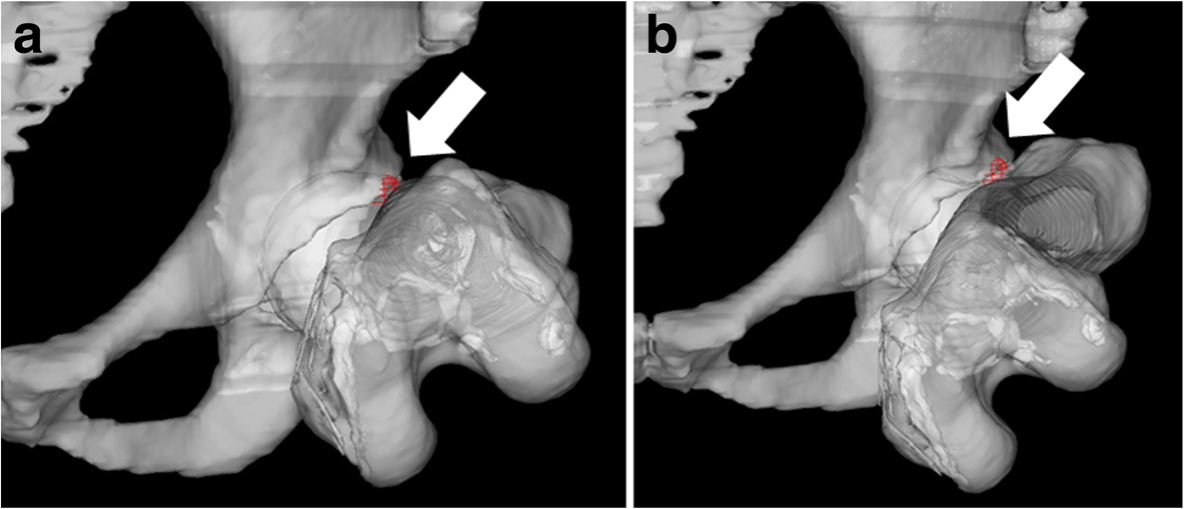
Pre- and postoperative 3D images of the impingement simulation Impingement points are identified by arrows. The impingement point at the femoral head-neck junction at 90° flexion and maximum internal rotation of the hip joint is shown preoperatively (**a**) and postoperatively (**b**)

ZedHip® (LEXI Co., Ltd., Tokyo, Japan) software was used to perform impingement simulation analysis. Digital imaging and communication in medicine (DICOM) data for each patient were transferred to ZedHip®, and three-dimensional (3D) simulation models of the pelvis and femur were constructed. The functional pelvic plane in a supine position was used as the pelvic plane reference. To set the femoral plane reference, the femoral head center was defined by assigning four reference points. The axis was set using two reference points: the head center and the mid-point between the medial and lateral epicondyles. Then, the pelvis and femur were segmented. The bony impingement point on the femoral head-neck junction at 90° flexion and maximum internal rotation of the hip joint was then identified (Fig. 1). The bony impingement was contact point between the acetabular rim and the femoral head-neck junction at the terminal point of the impingement simulation. If the impingement point appeared below 90° of flexion, the case was excluded. The impingement region of interests was defined into 2 regions (i.e., proximal region and distal region) in A-P view of the hip joint [17], and difference in the distribution of the impingement point was evaluated between cam-FAI and DDH W/ cam groups.

**Fig. 2:**
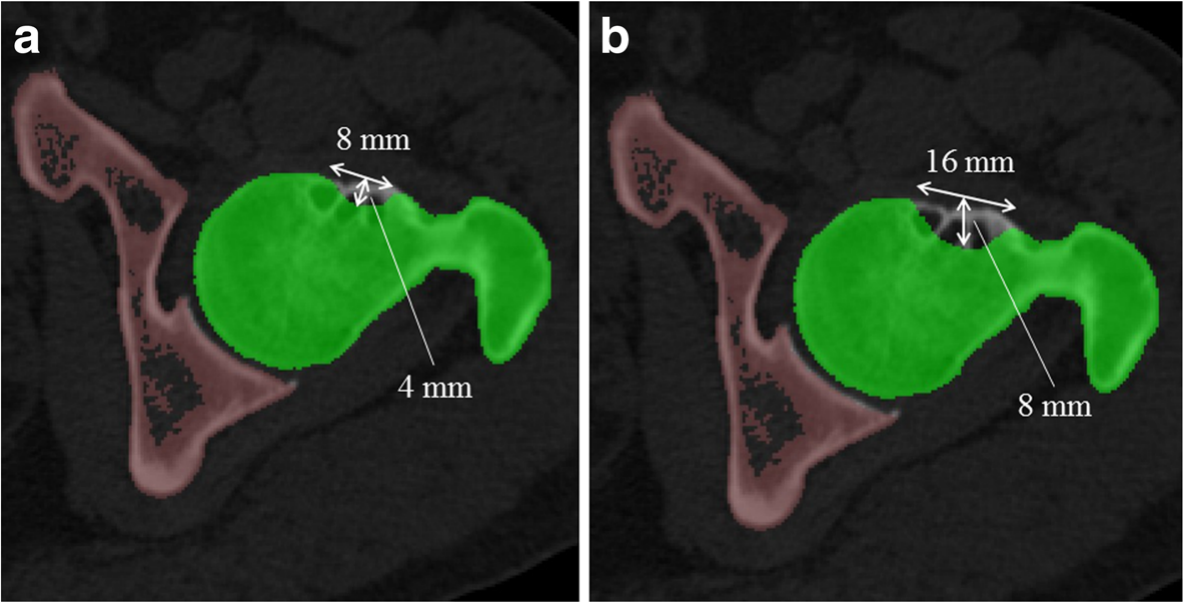
The virtual resection of the impingement point The depth and width of the resection in the horizontal slice and the vertical resection length around the impingement point were standardized (i.e., slight resection (**a**), sufficient resection (**b**))

Virtual resection of the impingement point was then performed by the same investigator using ZedHip®. The center of resection area was decided based on the impingement point by ZedHip®. Figure 2 shows the defined area of resection based on each impingement point. Because the resection depth for cam deformity would typically be 4–8 mm [18], two different versions were modeled: slight resection at a depth of 4 mm and sufficient resection at a depth of 8 mm. The width of the resected area in a horizontal slice was modeled as slight (8 mm) and sufficient (16 mm). The length of the resection was standardized at 15 mm (1.5 mm slice × 10 slices) in each axial image [19]. The edge of each resection area was trimmed smoothly to simulate clinical situation.

The maximum flexion angle and maximum internal rotation angle at 90° flexion was measured both before and after virtual osteochondroplasty using ZedHip® using each resection model (i.e., slight resection and sufficient resection) for cam-type FAI, borderline DDH with cam deformity, and borderline DDH without cam deformity cases.

### Statistical analysis

The demographic data and imaging study results for all groups were evaluated using one-way factorial analysis of variance, as were the maximum flexion angle and maximum internal rotation angle at 90° flexion before virtual osteochondroplasty and improvements in the angle of maximum flexion angle and maximum internal rotation at 90° flexion. Difference in the distribution of the impingement point was evaluated using contingency table. Intraobserver reliability was calculated using intraclass correlation coefficients and their 95% confidence intervals (CIs) to assess the reliability of the improvement in the internal rotation angle at each resection models. *P* values < 0.05 were considered statistically significant. All statistical analyses were performed using dedicated statistical analysis software (SPSS 16.0; IBM Corp., Armonk, NY, USA).

## Results

The demographic data for the three groups are presented in the Table 1. The mean body mass index (BMI) in the cam-FAI group was significantly higher than that in the DDH W/O cam group (*P* = 0.012). positive crossover sign was seen in 9 hips out of 38 hips, and it was seen most in cam-type FAI group.

**Table 1:** Demographic data for the cam-FAI, DDH W/ cam, and DDH W/O cam groups

	Cam-FAI (*n* = 15)	DDH W/ cam (*n* = 11)	DDH W/O cam (*n* = 12)
Sex (male / female)	11 / 4	1 / 10	1 / 11
Age (mean ± SD)	48.7 ± 12.5	49.9 ± 11.9	44.8 ± 15.1
BMI (mean ± SD)	23.5 ± 3.2	22.1 ± 2.5	20.4 ± 2.2
CE angle (mean ± SD)	32.3 ± 4.2	22.0 ± 1.5	21.7 ± 1.7
alpha angle (mean ± SD)	65.7 ± 7.6	63.6 ± 6.1	49.5 ± 3.6
Tönnis grade (0 / 1)	10 / 5	7 / 4	10 / 2
Crossover sign (positive/ negative)	6 / 9	2 / 9	1 / 11

*BMI* body mass index, *CE* center-edge, *SD* standard deviation

**Fig. 3:**
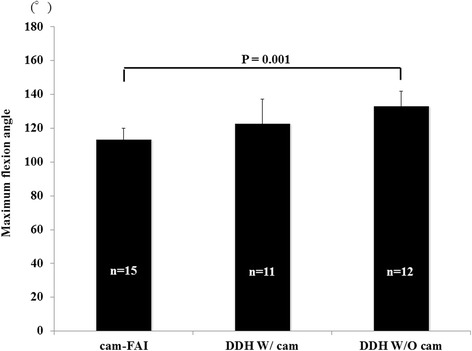
Maximum flexion angle in the three groups before virtual osteochondroplasty The mean maximum flexion angle in the DDH W/O cam group was significantly greater than that in the cam-FAI FAI group (*P* = 0.001)

**Fig. 4:**
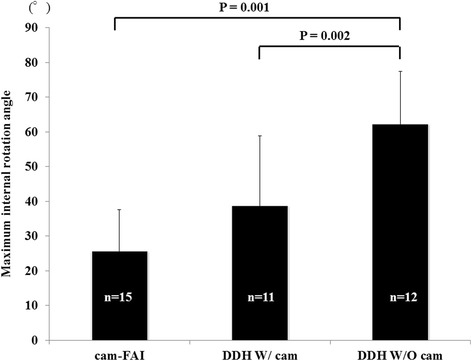
Maximum internal rotation angle in the three groups before virtual osteochondroplasty The mean maximum internal rotation angle in the DDH W/O cam group was significantly greater than that in the cam-FAI (*P* = 0.001) and DDH W/ cam groups (*P* = 0.002)

The differences in the maximum flexion angle prior to virtual osteochondroplasty are shown in Fig. 3. The mean maximum flexion angle in the cam-FAI group was 113.1 ± 6.9°, whereas that in the DDH W/ cam and DDH W/O cam groups was 122.5 ± 14.7° and 132.8 ± 9.0°, respectively. The mean maximum flexion angle in the DDH W/O cam group was significantly greater than that in the cam-FAI group (*P* = 0.001). Differences in the maximum internal rotation angle prior to virtual osteochondroplasty are shown in Fig. 4. The mean maximum internal rotation angle in the cam-FAI group was 25.8 ± 11.8°, whereas that in the DDH W/ cam and DDH W/O cam groups was 38.9 ± 19.9° and 62.4 ± 15.0°, respectively. The mean maximum internal rotation angle in the DDH W/O cam group was significantly greater than that in the cam-FAI (*P* = 0.001) and DDH W/ cam groups (*P* = 0.002).

The impingement region in the cam-FAI group was distributed to 9 hips in the proximal region, and 6 hips in the distal region. The impingement region in the DDH W/ cam group was distributed to 8 hips in the proximal region, and 3 hips in the distal region. There was no significant difference in the distribution of the impingement point between cam-FAI and DDH W/ cam groups (*P* = 0.68).

**Fig. 5:**
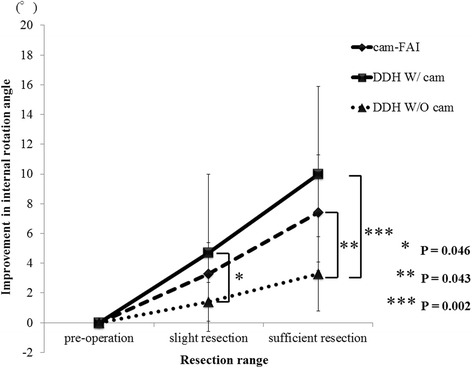
Improvement in the internal rotation angle after each type of resection The mean improvement in the internal rotation angle in the DDH W/ cam group after slight resection was significantly greater than that in the DDH W/O cam group (*P* = 0.046), as was that after sufficient resection (*P* = 0.002). The improvement in the cam-FAI group was significantly greater than that in the DDH W/O cam group (*P* = 0.043)

**Fig. 6:**
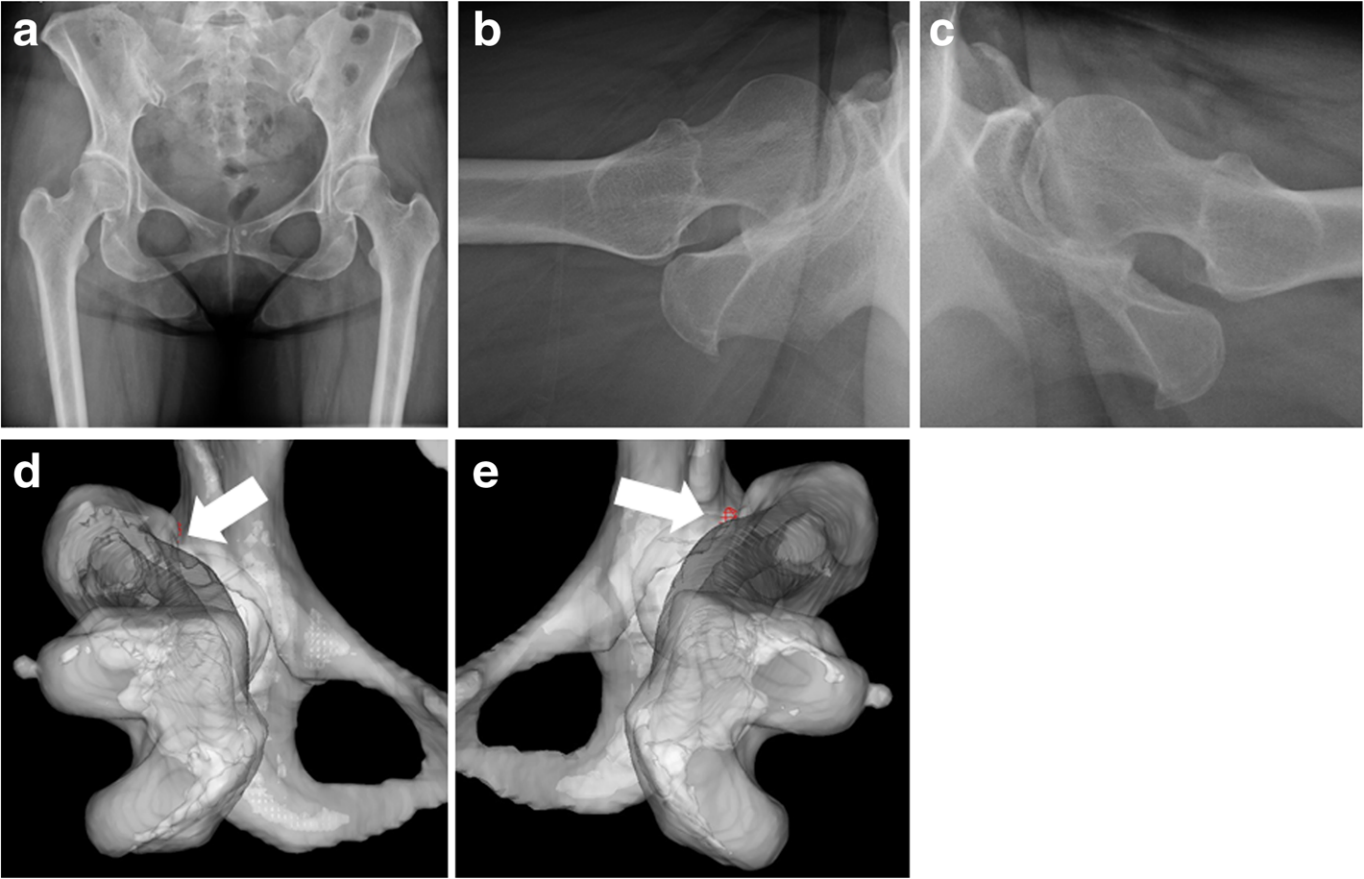
A representative case of borderline DDH These images are of a 47-year-old woman with borderline DDH with cam deformity in the right hip and borderline DDH without cam deformity in the left hip. The CE angle in the right hip was 21° and that in the left was 24° (**a**). The alpha angle in the right hip was 67° (**b**) and that in the left was 51° (**c**). Impingement points are identified by arrows (**d, e**). The preoperative maximum internal rotation angle for both hip joints was 49°. The internal rotation angle in the right hip after slight resection was 53°, whereas that after sufficient resection was 65° (**d**). The final improvement in the internal rotation angle of the right hip joint was 16°. However, the internal rotation angle in the left hip after sufficient resection was 52° (**e**). Thus, the final improvement in the internal rotation angle of the left hip joint was 3°

The mean improvement in the flexion angle in the cam-FAI group after slight resection was 0.3 ± 1.0°, whereas that in the DDH W/ cam and DDH W/O cam groups was 0.6 ± 1.3° and 0.0 ± 0.0°, respectively. The mean improvement in the flexion angle in the cam-FAI group after sufficient resection was 0.4 ± 1.5°, whereas that in the DDH W/ cam and DDH W/O cam groups was 1.2 ± 2.0° and 0.2 ± 0.6°, respectively. There was no significant difference in any of these values between the three groups, regardless of resection type. The mean improvement in the internal rotation angle in each of the three groups after virtual resection is shown in Fig. 5. The improvement in the cam-FAI group after slight resection was 3.3 ± 2.1°, whereas that in the DDH W/ cam and DDH W/O cam groups was 4.7 ± 5.3° and 1.4 ± 1.3°, respectively. The mean improvement in the internal rotation angle after slight resection was significantly greater in the DDH W/ cam group than in the DDH W/O cam group (*P* = 0.046); however, there was no significant difference between the cam-FAI and DDH W/O cam groups. The mean improvement in the internal rotation angle in the cam-FAI group after sufficient resection was 7.4 ± 3.9°, whereas that in the DDH W/ cam group and DDH W/O cam groups was 10.0 ± 5.9° and 3.3 ± 2.5°, respectively. The mean improvement in the internal rotation angle in the DDH W/ cam group after sufficient resection was significantly greater than that in the DDH W/O cam group (*P* = 0.002), as was that in the cam-FAI group (*P* = 0.043). There was no significant difference between the cam-FAI and DDH W/ cam groups in the improved internal rotation angle regardless of resection type, however, that in the DDH W/ cam group was tended to show more improvement than cam-FAI group for both slight and sufficient resections (Fig. 6).

In assessing the reliability of measuring the improvement in the internal rotation angle by computer simulation, intraobserver reliability was 0.917 (95% CIs, 0.792–0.969) at slight resection, and that was 0.828 (95% CIs, 0.594–0.933) at sufficient resection, both showing good reliability.

## Discussion

Here, we demonstrated that patients with borderline DDH with cam and cam-type FAI showed similar and significant improvement in the internal rotation angle after virtual osteochondroplasty; however, the procedure was not effective for those with borderline DDH without cam. The clinical implication is that it may be better to consider performing osteochodroplasty even in borderline DDH with cam deformity. This is the first study to use computer simulation analysis to examine the effect of cam osteochondroplasty in cases of borderline DDH with cam deformity. In fact, there was no significant difference of pre-operative internal rotation angle between cam-FAI and DDH W/ cam groups. Although preoperative internal rotation angle between DDH W/ cam group seems to be good enough to avoid impingement, the virtual osteochondroplasty resulted in the most improvement in three groups.

Several studies have performed virtual resection of cam lesions using 3D finite element models and computer simulations. Alonso-Rasgado et al. [18] reported that, to reduce the risk of femoral neck fracture, the resection depth should be < 10 mm or 1/3 the diameter of the neck, whereas Rothenfluh et al. [19] reported that the depth of the resection should be no more than 20% of the diameter of the femoral neck, and that the resection length should be no more than 20 mm. The main aim of these studies was to use 3D finite element analysis to measure mechanical strength after virtual osteochondroplasty. Bedi et al. [20] used computer simulation models to examine improvements in ROM after performing virtual cam and pincer resection for FAI. While virtual osteochondroplasty at defined regions of impingement resulted in a significant improvement in both hip flexion and internal rotation angle in the eight FAI cases examined, no patients with borderline DDH were included. Nevertheless, these previous simulation studies using virtual osteochondroplasty showed that computer simulation methods have an important role to play in predicting surgical outcome. We believe that the novelty of the present study is the finding of differences in surgical outcome, i.e., improved ROM after virtual osteochondroplasty, for patients with one of three different conditions: cam-type FAI, borderline DDH, and especially borderline DDH with cam deformity.

Previous studies examined clinical results after hip arthroscopy in cases of DDH [13, 21–23]. Byrd et al. [13] and Jayasekera N et al. [22] reported satisfactory results, even in the presence of dysplasia. Similarly, Fukui et al. [21] reported that arthroscopy of the hip can be successful in young patients with mild to moderate DDH or FAI. However, Uchida et al. [23] reported a clinical failure rate of 32.1% when treating patients with DDH. Ida et al. [7] reported that 40% of patients with DDH showed radiographic evidence of cam deformity, and that significantly more patients in the DDH with cam deformity group than in the DDH alone group had a positive preoperative anterior impingement test. Paliobeis et al. [8] reported that 47% of patients with FAI showed radiographic evidence of dysplasia. Thus, co-existence of borderline DDH with cam deformity appears certain. Based on the results presented herein, cam osteochondroplasty may be an effective treatment for cases of borderline DDH with cam deformity, even if they undergo only slight resection, whereas cases of cam-type FAI should undergo sufficient resection. By contrast, the procedure would appear to be ineffective for those with borderline DDH cases without cam deformity. Thus, it is essential to establish the co-existence of cam deformity, especially in cases of borderline DDH. In actual surgery, the joint capsule must be released to expose femoral head-neck junction to perform cam resection. In that case, the plication of the capsule after cam resection is essential. These procedures are relatively complicated and needs certain invasion. If there is no need to perform the cam resection, capsular release could be minimized. Therefore, it is important to predict whether the cam resection is effective or not preoperatively by virtual osteochondroplasty. While hip arthroscopy for severe DDH cases should be cautious [21, 23], performing the computer simulation of virtual osteochondroplasty may provide the positive reason for the cam resection in borderline DDH with cam deformity cases.

One of the limitations of this study is that we were unsure whether our models of osteochondroplasty were optimized for cam deformity in terms of resection depth, width, and length of the femoral head-neck junction. Several studies reported “reasonable” resection areas for the femoral neck that would minimize the risk of femoral neck fracture; therefore, we were guided by these studies [18, 19]. While the clinical validity of this resection model needs to be considered, it was essential that we used standardized models of osteochondroplasty for computer simulation. Another limitation is that the influence of soft tissues, including the labrum, ligaments, and joint capsule, was not considered in the computer simulation. Further, the clinical important issue is that we could not measure actual clinical outcome after osteochodroplasty in all cases. The relationship between actual clinical outcomes and virtual surgery should be investigated. Regarding this point, we should note that it is impossible to argue the clinical outcomes only by improvement of ROM. However, Kemp et al. [24] reported that greater hip ROM was independently associated with better scores in several clinical outcomes including quality of life. Thus, the improvement of ROM should be one of the positive factors for getting satisfactory clinical outcomes.

## Conclusion

In conclusion, virtual osteochondroplasty resulted in a significant improvement in internal rotation angle in cases of borderline DDH with cam deformity but not in cases of borderline DDH without cam deformity. Thus, careful consideration should be given to cam resection in cases of borderline DDH with cam deformity using computer simulation. Further studies are needed to validate the clinical outcome of patients with borderline DDH with cam deformity that undergo osteochondroplasty.

## Abbreviations

3D: Three-dimensional
A-P: Antero-posterior
BMI: Body mass index
CE: Center-edge
CIs: Confidence intervals
CT: Computed tomography
DDH: Developmental dysplasia of the hip
DICOM: Digital imaging and communication in medicine
FAI: Femoroacetabular impingement
ROM: Range of motion
